# Effectiveness of corticosteroids for post-extubation stridor and extubation failure in pediatric patients: a systematic review and meta-analysis

**DOI:** 10.1186/s13613-020-00773-6

**Published:** 2020-11-18

**Authors:** Satoshi Kimura, JiYoon B. Ahn, Mai Takahashi, Sohee Kwon, Stefania Papatheodorou

**Affiliations:** 1 Department of Epidemiology, Harvard T.H. Chan School of Public Health, 677 Huntington Avenue, Boston, MA 02115 USA; 2 Department of Pediatric Intensive Care Unit, The Royal Children’s Hospital, 50 Flemington Rd, Parkville, VIC 3052 Australia; 3 Department of Internal Medicine, Mount Sinai Beth Israel, 317 E 17th St, New York, NY 10003 USA

**Keywords:** Mechanical ventilation, Breathing, Laryngeal edema, Respiratory sounds, Glucocorticoids, Pediatrics

## Abstract

**Background:**

While the results of previous meta-analyses have shown beneficial effects of corticosteroid therapy on post-extubation stridor and extubation failure in adults, these results might not be generalizable to children because of the differences in anatomy and structure. We aimed to determine the benefits of corticosteroids on those outcomes in pediatric populations.

**Methods:**

We searched PubMed, EMBASE, and reference lists of articles from inception until February 2019. Randomized controlled trials and observational studies on the efficacy of systemic corticosteroid administration given prior to elective extubation in mechanically ventilated pediatrics were eligible. Outcomes included post-extubation stridor indicating laryngeal edema and extubation failures.

**Results:**

A total of ten randomized controlled trials with 591 pediatric patients were included: seven of the ten studies for post-extubation stridor/suspected upper airway obstruction and nine of the ten studies for extubation failure. The estimate of pooled odds ratios (ORs) for post-extubation stridor/suspected upper airway obstruction was 0.40 (95% CI: 0.21–0.79). When analysis was restricted to trials that had explicit data for infants and explicit data for pediatric patients under 5 years old excluding infants, the estimates of pooled ORs were 0.53 (95% CI: 0.20–1.40) and 0.68 (95% CI: 0.38–1.22), respectively. For pediatric patients who received corticosteroids, there was a 0.37-fold lower odds of extubation failure than that in pediatric patients who did not receive corticosteroids (OR, 0.37; 95% CI, 0.22–0.61). While three observational studies were included in this review, their estimates have a potential for bias and we did not perform a meta-analysis.

**Conclusions:**

Despite a relatively small sample size in each randomized controlled trial and wide ranges of ages and steroid administration regimens, our results suggest that the use of corticosteroids for prevention of post-extubation stridor and extubation failure could be considered to be acceptable in pediatric patients.

## Background

Laryngeal edema can cause acute upper airway obstruction and is one of the life-threatening sequelae associated with mechanical ventilation under endotracheal intubation. Mechanical pressure or irritation from the endotracheal tube is associated with the potential development of laryngeal edema, resulting in stridor upon extubation [1, 2]. The incidence of post-extubation laryngeal edema in critical care patients, characterized by stridor and/or respiratory distress, ranges from 4–37% depending on the population being investigated [3–8]. Post-extubation laryngeal edema increases the probability of reintubation, leading to nosocomial pneumonia, a prolonged duration of endotracheal intubation, a longer duration of stay in the intensive care unit (ICU), and high mortality [9, 10].

Post-extubation edema or stridor following endotracheal intubation is more commonly symptomatic in children than in adults due to differences in airway anatomy [1, 7, 11]. Compared with adults, the submucosal connective tissue in the subglottic area is looser in children, allowing fluid to accumulate [1]. In addition, the less expandable cricoid cartilage ring around the trachea and the smaller size of the larynx in children make the effect of reduction in airway diameter, even a small amount of edema, more likely to result in infringement of the cross-sectional area of the airway and large airway resistance, causing more severe airflow limitation [1, 2].

There have been many studies on the effectiveness of intravenous corticosteroids for preventing or treating this life-threatening complication. Theoretically, corticosteroids may be effective due to their anti-inflammatory effects for reducing laryngeal infiltration of inflammatory cells and for reducing edema [2, 12, 13]. Recently, meta-analyses have shown beneficial results of corticosteroid therapy for prevention of post-extubation stridor and reintubation in adults [2, 14, 15]. However, evidence for the benefits of the administration of steroids in adults may not be generalizable to neonates or children because of the differences in anatomy in pediatric patients. In this meta-analysis, we aimed to determine the effects of corticosteroids, comparing to no corticosteroid use, on laryngeal edema possibly resulting in post-extubation stridor or other findings indicating laryngeal edema and extubation failure in a pediatric population (neonates, infants, children or adolescents) and to determine potential adverse effects of corticosteroids in this population.

## Methods

### Literature search

We searched PubMed and EMBASE from inception until February 2019 by using MESH terms and synonyms of “laryngeal edema”, “extubation” and “corticosteroids”. We also reviewed the references of retrieved articles for potentially eligible trials. The electronic search strategies are shown in Appendix 1.

### Eligibility criteria

We followed the Preferred Reporting Items for Systematic Reviews and Meta-Analyses (PRISMA) statement to select relevant studies for our research [16]. We included randomized controlled trials (RCTs) and observational studies on the efficacy of systemic corticosteroid administration given prior to elective extubation in mechanically ventilated pediatrics (neonates, infants, children or adolescents). We excluded trials in which corticosteroids were administered only after extubation, trials in which nebulized corticosteroids were used, trials in which a comparison was made with other interventions, trials that included patients with potentially steroid-effective diseases such as croup, asthma, and respiratory syncytial virus infection, and trials for which reports were not written in English.

### Data collection

We used the online Covidence software [17] for screening, selection, and data extraction. All records were independently evaluated by two members of the authors’ team (SK, JA) through title and abstract screening. Disagreements regarding title and abstract eligibility were resolved by a discussion between the two members as well as a third author (MT). Full text screening of selected papers was conducted by two members of the authors’ team (SK, JA). Disagreements regarding record eligibility were resolved by a discussion between the two authors and a third author (MT) cast a final vote if a consensus was not reached. All studies meeting the inclusion criteria were retained in the analysis. Access to all full texts could be obtained without having to contact corresponding authors. Two authors also independently extracted the following data from each study included in our analysis: first author, publication year, location, study setting, sample size, gender, age, regimen of corticosteroid administration, observational duration, and frequencies of manifestations related to laryngeal edema such as stridor and reintubation. When available, we recorded data for adverse events such as hyperglycemia, hypertension, infection, and gastrointestinal bleeding. Corticosteroid doses were standardized to hydrocortisone equivalents, according to the relative anti-inflammatory potency of each drug: 25 mg hydrocortisone being equivalent to 5 mg methylprednisolone or 1 mg dexamethasone [14].

We assessed the risk of bias using the Cochrane risk of bias assessment tool for RCTs [18]. We also assessed the risk of bias using the Newcastle–Ottawa Scale for nonrandomized studies [19], considering studies achieving 6 points or more as high quality [20]. In addition to the scale, we emphasized comparability between groups because of the importance for validity of observational studies. Any conflicts or disagreements in the whole process were solved by discussion and consensus.

### Outcomes and data analysis

The primary outcome was post-extubation stridor/suspected upper airway obstruction indicating laryngeal edema related to endotracheal intubation. Secondary outcomes were extubation failures defined as reintubation or use of non-invasive ventilation within 72 h after extubation and adverse effects related to corticosteroids. Binary outcomes were pooled by using a random-effect model for incidence of stridor/suspected upper airway obstruction in order to take clinical heterogeneity of the included populations and other sources of variation between studies into account. The DerSimonian and Laird random-effects model [21] was applied for the primary outcome with the odds ratio (OR) and 95% confidence interval (CI). A fixed-effect model with the Peto method [22] was used for the incidence of extubation failure to address rare events.

We evaluated heterogeneity between studies with the Cochrane *χ*^2^ test [23] and the *I*^2^ statistic with an *I*^2^ value higher than 50% indicating at least moderate heterogeneity [24]. Publication bias was assessed by visual inspection of the funnel plot and with the Egger test [25]. A sensitivity analysis for post-extubation stridor/suspected upper airway obstruction was performed by excluding trials in which stridor was used as one of the scores for upper airway obstruction, but other scores in the scoring system might be related to conditions other than laryngeal edema. Trials that used outcomes other than reintubation and additional airway manipulation as “extubation failure” and trials that included neonates with extremely low birth weights and in which corticosteroids were administered for more than 10 days for other purposes were excluded in a sensitivity analysis for extubation failure. Meta-regression was conducted for the associations of outcomes with cumulative dose of corticosteroid equivalent to hydrocortisone, timing of first corticosteroid treatment before extubation, and age. The statistical significance was set at *p* < 0.05 two-sided. All statistical analyses were performed using R 3.6.0 (R foundation for Statistical Computing, Vienna, Austria).

## Results

### Overviews of randomized controlled trials and risk of bias assessment

**Fig. 1:**
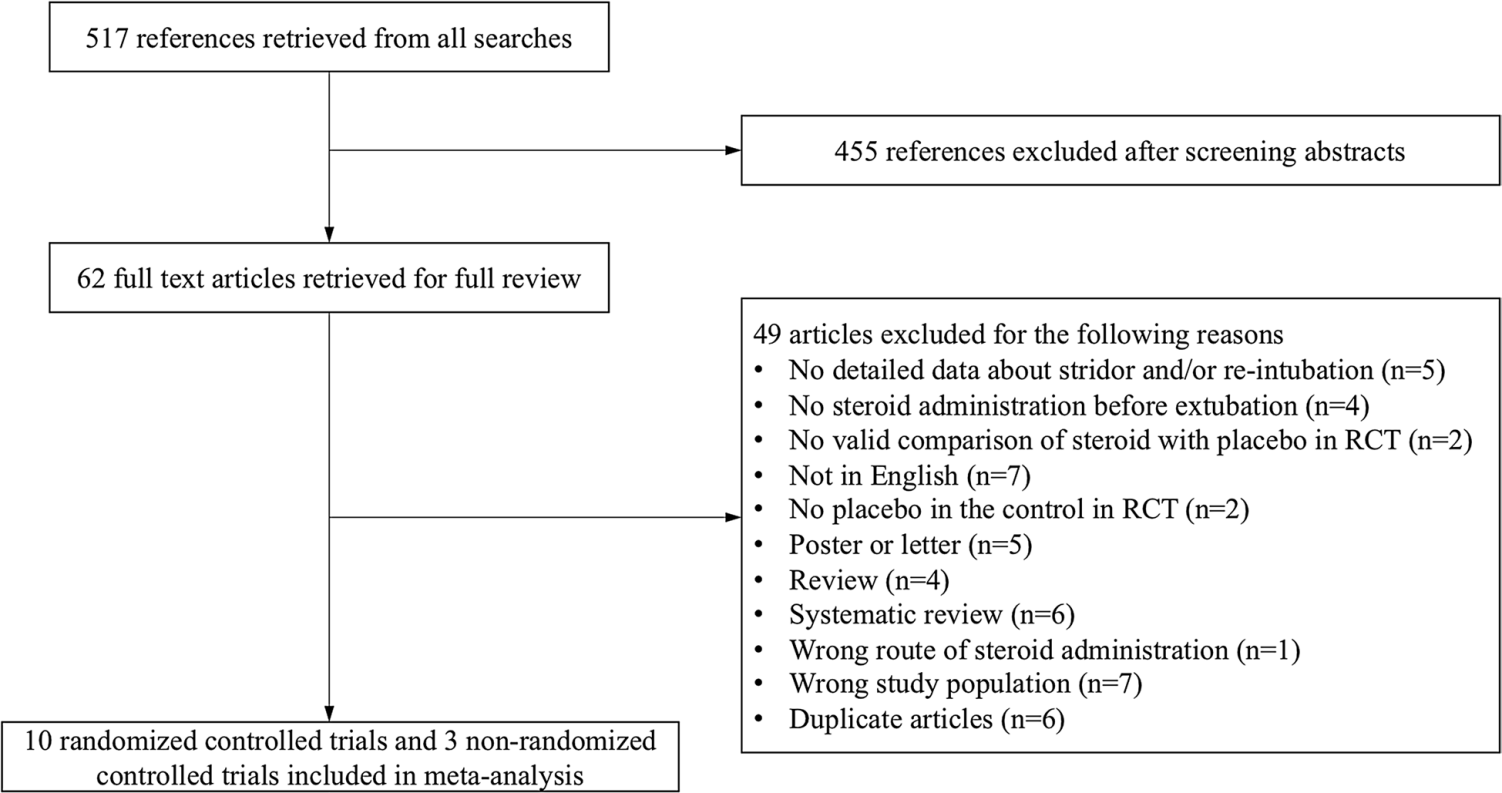
Study selection workflow

The search retrieved a total of 517 references. After application of the inclusion and exclusion criteria, 10 RCTs were included in this review. A PRISMA flowchart of this process is shown in Fig. 1. Details of the included RCTs are shown in Additional file 1: Table S1. There was one study restricted to neonates [26] and three studies included only infants [27–29], but the rest of the studies included pediatric patients with a wide range of ages. One study included participants with known laryngeal edema [30], six studies included patients at high risk for laryngeal edema such as patients with prolonged intubation and mechanical ventilation [11, 27, 28, 31–33], and three studies included participants to whom steroids were given for another reason such as expectation of improved pulmonary function [26, 29, 34]. Dexamethasone was used in nine studies [11, 26–33] and methylprednisolone was used in one study [34] for intervention. There was a range of different regimens of dosing, frequency, and timing for administration of corticosteroids among the trials (Additional file 1: Table S1).

**Fig. 2:**
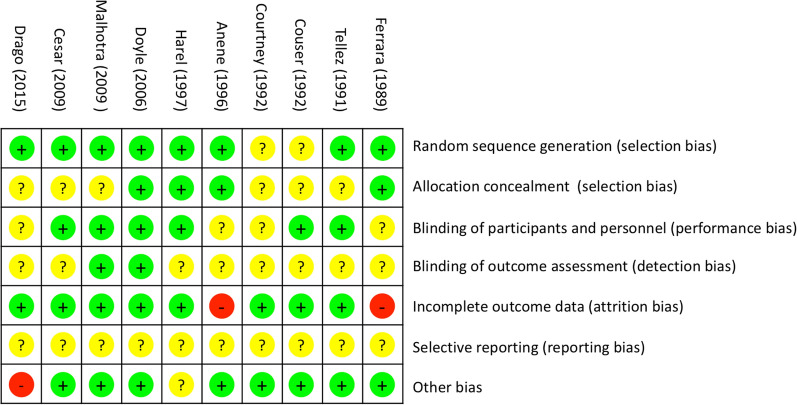
Risk of bias summary of randomized controlled trials

Eight (80%) out of ten trials and four (40%) out of ten trials had a low risk of bias in random sequence generation and allocation concealment, respectively. Reports of two trials explicitly mentioned both blinding of participants and personnel and blinding of outcome assessors. Eight trials showed a low risk of bias in incomplete outcome data. A summary of the risk of bias assessment for the RCTs is shown in Fig. 2.

### Overview of observational studies and risk of bias assessment

After application of the inclusion and exclusion criteria, three observational studies were included in this review [35–37]. Details of the observational studies are shown in Additional file 1: Table S2. One of the studies was a prospective cohort study [37] and there were two retrospective cohort studies [35, 36].

All of the observational studies showed a Newcastle–Ottawa Quality score of 6, which is classified as “high quality” (Additional file 1: Table S3). However, in those studies, the relationship between corticosteroids and post-extubation stridor was estimated by just comparing two groups without adjustment for confounders. Furthermore, as shown in Additional file 1: Table S2, only adjustment for age was made in one study and there was no adjustment for any potential confounders in the other two studies. Given that the groups were not comparable in their baseline characteristics, their estimates have a potential for bias. Therefore, we did not perform a meta-analysis.

### Post-extubation stridor/suspected upper airway obstruction

**Fig. 3:**
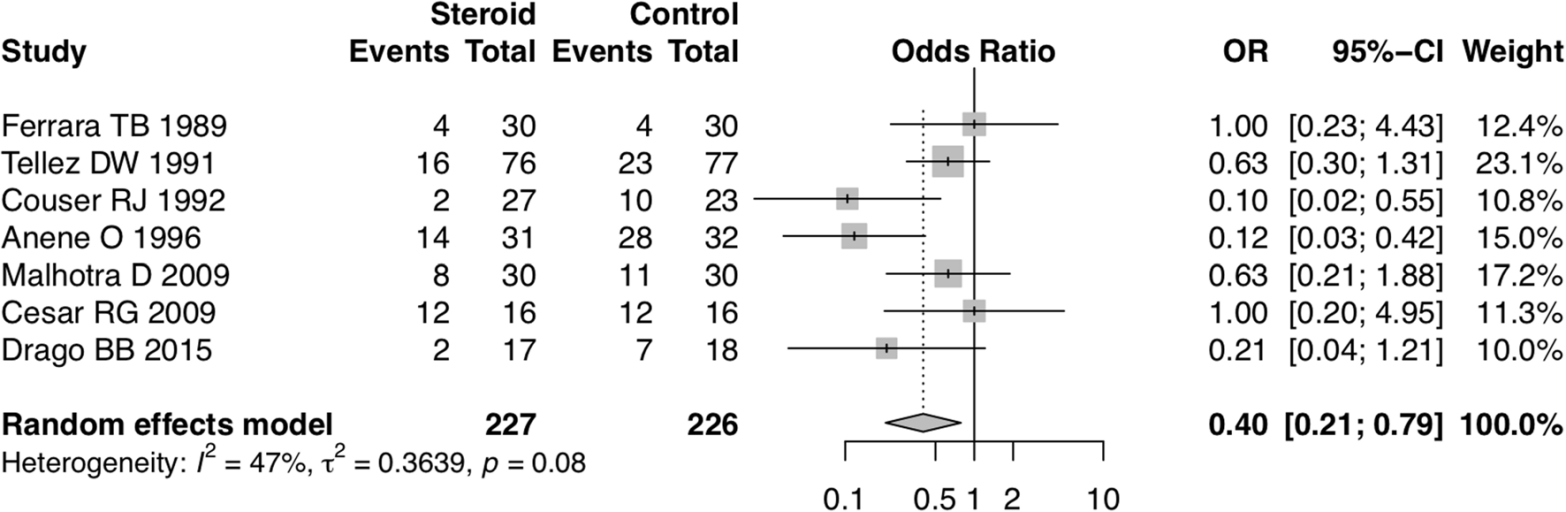
Result of meta-analysis for effects of corticosteroids on incidence of stridor/suspected upper airway obstruction in randomized controlled trials. An odds ratio (OR) less than 1 favors corticosteroid administration

Reports for seven of the 10 RCTs, including 453 patients, showed the incidence of stridor/suspected upper airway obstruction after extubation [11, 27, 28, 31–34]. The estimate of pooled ORs for post-extubation stridor was 0.40 (95% CI: 0.21–0.79, *I*^2^ = 47%; *p* = 0.08), indicating significantly lower odds of stridor with the use of corticosteroids (Fig. 3). Publication bias was not assessed by the funnel plot and the Egger test because of the small number of studies. In sensitivity analysis, including six trials that used stridor as a main assessment [11, 27, 28, 32–34], the estimate of pooled ORs for post-extubation stridor was 0.36 (95% CI: 0.17–0.74, *I*^2^ = 51%; *p* = 0.07) (Additional file 2 Figure S1A).

When analysis was restricted to trials with data for infants [27, 28, 31, 32], the estimate of pooled ORs was 0.53 (95% CI: 0.20–1.40, *I*^2^ = 41%; *p* = 0.16) (Additional file 2: Figure S1B). When analysis was restricted to trials with data for pediatrics under five years old, not including infants [31, 32], the estimate of pooled ORs was 0.68 (95% CI: 0.38–1.22, *I*^2^ = 0%; *p* = 0.75) (Additional file 2: Figure S1C). It was not feasible to conduct other age-subgroup analyses because most of the studies included a wide range of age groups and did not have accurate data for the incidence based on age categories.

### Extubation failure

**Fig. 4:**
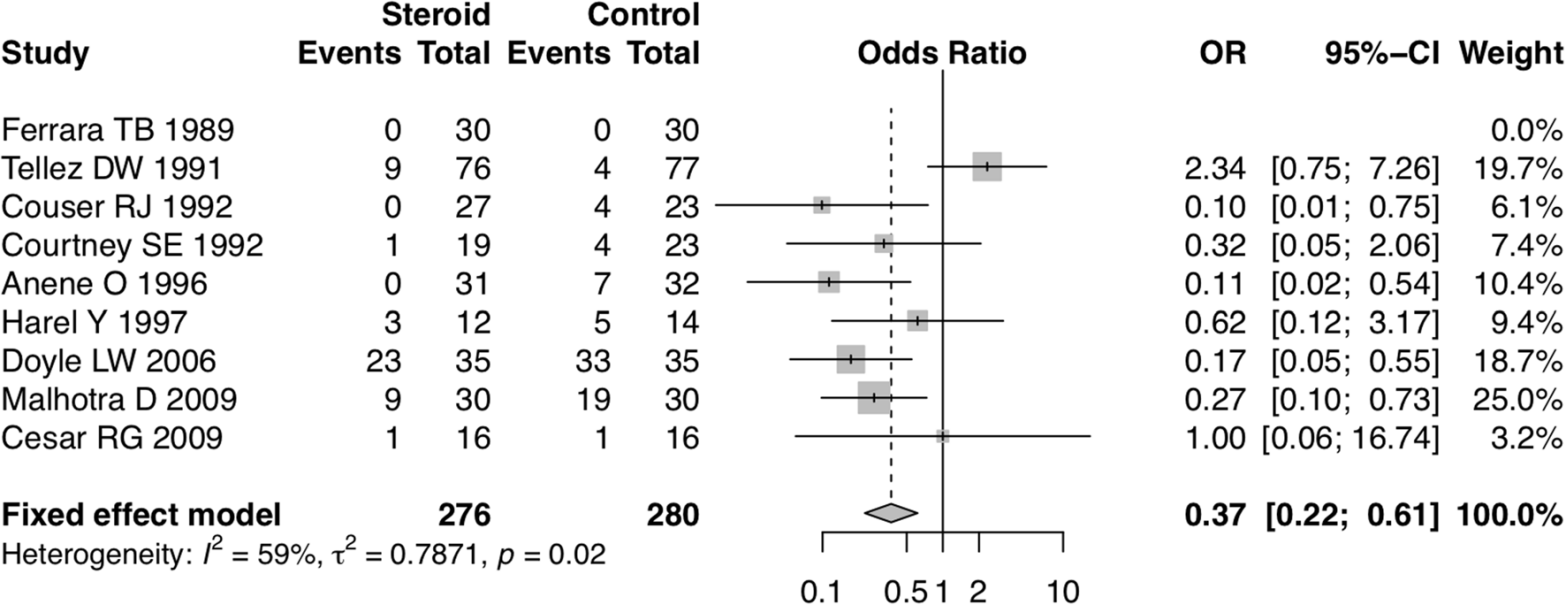
Result of meta-analysis for effects of corticosteroids on incidence of extubation failure in randomized controlled trials. An odds ratio (OR) less than 1 favors corticosteroid administration

Reports for nine of the 10 RCTs, including 553 patients, showed the incidence of extubation failure [11, 26–33]. Pediatric patients who received corticosteroids had a 0.37-fold lower odds of extubation failure than did those who did not receive corticosteroids (OR, 0.37; 95% CI, 0.22–0.61; *I*^2^ = 59%; *p* = 0.02) (Fig. 4). Publication bias was not assessed by the funnel plot and the Egger test because of the small number of studies. In sensitivity analysis, excluding one trial that included patients with acute respiratory distress syndrome and in which death and withdrawal from the study, as well as reintubation, were defined as extubation failure [26], the estimate of pooled ORs was 0.45 (95% CI: 0.26–0.80, *I*^2^ = 52%; *p* = 0.05).

When restricting analysis to trials with data in infants in the sensitivity analysis [27–29], the estimate of pooled ORs for extubation failure was 0.16 (95% CI: 0.03–0.92, *I*^2^ = 0%; *p* = 0.52) (Additional file 3: Figure S2). Other age-subgroup analyses could not be conducted because patients with a wide range of ages were included in each study.

### Adverse effects

Potential adverse effects of corticosteroids were infrequently assessed in the RCTs. Among them, hyperglycemia was the most frequently assessed potential adverse effect of corticosteroids, followed by gastrointestinal bleeding, infection, hypertension, and glycosuria (Table 1). Because of the small sample size and infrequent reporting of adverse effects of corticosteroids, we could not robustly assess the adverse effects of corticosteroids for pediatric patients.

**Table 1:** Adverse events reported as potentially attributable to corticosteroids

Adverse event	Author	Control	Steroids
Gastrointestinal bleeding	Anene et al. [33]	0/32	1/32
Hyperglycemia	Anene et al. [33]	0/32	0/32
	Drago et al. [34]	8/18	10/17
	Doyle et al. [26]	No significant differences in blood glucose concentration	
	Courtney et al. [29]	Mean serum glucose 87 mg/dl	Mean serum glucose 101 mg/dl
Infection	Drago et al. [34]	5/18	1/17
	Courtney et al. [29]	1/23	1/19
Hypertension	Anene et al. [33]	1/32	1/32
	Doyle et al. [26]	No significant differences in blood pressure	
Glucosuria	Couser et al. [27]	0/23	7/27

### Meta-regression analysis

In meta-regression analysis for the RCTs that provided enough evidence, [11, 26–34], we assessed whether there were a linear relationships between the effect size and covariates including cumulative corticosteroid dose, time from the first administration to extubation, and mean (or median) age. As shown in Additional file 1: Table S4, each coefficient of those covariates did not have statistical significance.

## Discussion

The effects of corticosteroids on laryngeal edema possibly resulting in stridor after and on extubation failure in pediatric patients were assessed in this study. Although there were wide ranges of corticosteroid regimens, cumulative doses, and frequencies and durations of administration among those trials, our meta-analyses demonstrated that corticosteroids significantly reduce the rates of post-extubation stridor/suspected upper airway obstruction and extubation failure when used in pediatric patients with or at risk of laryngeal edema. Although only a limited number of subgroup analyses for different age categories could be performed because each trial included children with various ages, the analyses showed neither a significant difference in odds of stridor for pediatric patients under five years old, not including infants, nor a significant difference in odds of extubation failure for infants.

In 2009, McCaffey et al. conducted the latest systematic review and meta-analysis, including children and adults, and concluded that corticosteroid administration is effective for preventing laryngeal edema (OR, 0.36; 95% CI, 0.27–0.49) and for reducing the incidence of extubation failure (OR, 0.56; 95% CI, 0.41–0.77) in critically ill patients of all ages [15]. However, there are some concerns about their conclusion. First, although the authors included three papers for neonates and four papers for pediatrics in their analyses, they combined those data with data for adults and analyzed the data together. Since the airway anatomy and structure are different for different ages, it may be inappropriate to analyze those data together. Second, they included a study conducted by Tibballs et al., which included children with croup [38]. Since croup is one of the diseases in which corticosteroids are effective, those patients should be excluded from a meta-analysis to assess the effect of corticosteroids on laryngeal edema. In the same year, a Cochrane Systematic Review [2] evaluated the effects of corticosteroids for these age groups separately. Two trials in neonates (*n* = 109) and two trials in children (*n* = 216) were included. The results of their meta-analyses showed that there was no statistically significant reduction in the rate of reintubation among neonates (risk ratio (RR), 0.10; 95% CI, 0.01–1.68) or among children (RR, 0.49; 95% CI, 0.01–19.65) and that there was no statistically significant reduction in the incidence of post-extubation stridor among neonates (RR, 0.42; 95% CI, 0.07–2.32). They only found a significant reduction in the incidence of post-extubation stridor among children with heterogeneity (RR, 0.53; 95% CI, 0.28–0.97; *I*^2^ = 53%). Based on these results, they concluded that the use of corticosteroids to prevent stridor after extubation has not been proven to be effective for neonates or children [2] and there has not been a consensus regarding the recommendation of corticosteroids for preventing post-extubation stridor in pediatric patients [39].

In order to reassess the effectiveness of corticosteroids for prevention of laryngeal edema in pediatric patients at this time, about 10 years after those meta-analyses were conducted [2, 15], we included three additional trials for stridor/suspected upper airway obstruction [11, 31, 34] (*n* = 453 in total, compared to 325 in the previous meta-analysis [2]; 39.4% increase in sample size). After excluding one study in which not only stridor, but also other elements for the scoring assessment that might not be related to upper airway obstruction were used [31], we obtained consistent results regarding the significant effect of corticosteroids. We also included five additional trials for extubation failure [11, 26, 29–31] (*n* = 556 in total, compared to 325 in the previous meta-analysis [2]; 71.1% increase in sample size). Trials for patients with respiratory distress disease were excluded in sensitivity analysis because corticosteroids might have an impact on pulmonary function and the incidence of reintubation, but there was still a significant effect on incidence of extubation failure. Our results suggest that the use of corticosteroids for prevention of post-extubation stridor and extubation failure should be considered to be acceptable in pediatric patients until clear evidence is provided by large-scale and appropriately conducted RCTs.

One of the main purposes of this review was to assess the effects of corticosteroids separately by age groups. Due to the lack of sufficient data and the wide range of age groups in each study, however, we could conduct only a limited number of subgroup analyses for infants and children under 5 years old. In these subgroup analyses, we did not find significant effect of corticosteroids on either outcome except for the effect of corticosteroids for extubation failure in infants. Because of the small sample size and/or high heterogeneity, however, it is difficult to reject the effect of corticosteroids for the other outcome or in different age group. In addition, there was also heterogeneity in the indication of corticosteroids among the selected population. Corticosteroids were given for patients with known laryngeal edema, patients at high risk for laryngeal edema, or another reason. Since it is difficult to conduct subgroup analyses considering both indication of corticosteroids and age, it is still unclear which population should receive corticosteroids before extubation.

Meta-regression analyses for stridor/suspected upper airway obstruction showed that cumulative dose of corticosteroids and time from the first dosing to extubation have negative coefficients in each model. Although they did not show statistical significance, the trends of more effectiveness for a larger amount of cumulative corticosteroid dose and for longer duration between the first dosing and planned extubation are consistent with results from a Cochrane Systematic Review conducted by Khemani et al., showing that corticosteroids are more effective with multiple doses administered 12–24 h prior to extubation [2]. A positive coefficient of age in meta-regressions could be interpreted as beneficial effects of corticosteroids for younger patients, but it was also not statistically significant.

Although our study provides the latest insight regarding this topic using rigorous methodology, this study also has some limitations. First, the sample sizes of the primary studies were relatively small and it is likely that some data for outcomes were not reported. However, exchangeability between the compared groups was demonstrated in the primary studies, and we can therefore assume that randomization was effective. Although the effects of corticosteroids on stridor and reintubation were statistically significant, sample sizes might have been too small to detect significant differences in the effects for different ages, different cumulative amounts of corticosteroids, and different durations between the first administration and extubation in the meta-regression analysis. In addition, it was not possible to conduct some subgroup analyses based on age groups in our study, and some analyses restricted to certain age groups failed to show significant effects of corticosteroids on both outcomes, possibly because of the small sample size. Second, the studies included in our meta-analysis differed in terms of age and corticosteroid regimens as shown in Additional file 1: Table S1 and there was statistical heterogeneity with relatively high *I*^2^ and *τ*^2^. Although the same intravenous corticosteroid was used in all but one of the studies, heterogeneity of age and detailed corticosteroid regimen makes it difficult to assess the dose–response effects of those medications based on the available data. In addition, possible difference of the type of tracheal tube, cuffed tube or uncuffed tube, in each patient and in each study could have different impact on the incidence and the severity of laryngeal edema, potentially contributing to the heterogeneity. We were not able to test for publication bias in our analysis because less than 10 studies were available, therefore the tests would be underpowered. Finally, because of the small sample sizes and infrequent reporting of adverse effects of corticosteroids, we could not robustly assess the adverse effects of corticosteroids for those pediatric patients.

## Conclusions

Our meta-analysis showed that the use of corticosteroids is associated with significant reductions in post-extubation stridor and extubation failure in pediatric patients. These findings need careful interpretations because of a relatively small number of sample size in each RCT and a wide range of ages and corticosteroid administration regimens. Larger and properly conducted RCTs are needed to verify our results and provide adequate evidence of the side-effects.

### Supplementary information

**Additional file 1: Table S1.** Summary of the characteristics of randomized controlled trials for assessment of post-extubation stridor and/or extubation failure. **Table S2.** Summary of the characteristics of observational studies for assessment of post-extubation stridor and/or extubation failure. **Table S3.** Quality assessment of observational studies. **Table S4.** Meta-regression analysis of post-extubation stridor and reintubation.

**Additional file 2: Figure S1.** (A) Result of meta-analysis for effects of corticosteroids on incidence of stridor in randomized controlled trials. Results of meta-analysis for effects of corticosteroids on incidence of stridor/suspected upper airway obstruction among (B) infants and (C) pediatrics under five years old, not including infants in randomized controlled trials. An odds ratio (OR) less than 1 favors corticosteroid administration.

**Additional file 3: Figure S2.** Results of meta-analysis for effects of corticosteroids on incidence of extubation failure among infants in randomized controlled trials. An odds ratio (OR) less than 1 favors corticosteroid administration.

## Data Availability

The datasets used and/or analysed during the current study are available from the corresponding author on reasonable request.

## Appendix 1: Search strategies

PubMed: ("Intubation, Intratracheal"[MeSH] OR "Laryngeal Edema"[Mesh] OR laryngeal edema[tiab] OR laryngeal oedema[tiab] OR (stridor*[tiab] AND (intubat*[tiab] OR extubat*[tiab] OR tracheal tube*[tiab]))) AND ("Glucocorticoids" [Pharmacological Action] OR "Glucocorticoids"[Mesh] OR steroid*[tiab] OR corticosteroid*[tiab] OR glucocorticoid*[tiab] OR prednisone[tiab] OR prednisolone[tiab] OR methylprednisolone[tiab] OR dexamethasone[tiab] OR cortisone[tiab] OR hydrocortisone[tiab] OR budesonide[tiab] OR fluticasone[tiab] OR ciclesonide[tiab] OR triamcinolone[tiab] OR beclomethasone[tiab] OR flunisolide[tiab] OR mometasone[tiab]) AND ("Infant"[mesh] OR "Child"[Mesh] OR "Adolescent"[mesh] OR "Pediatrics"[Mesh] OR "Intensive Care Units, Pediatric"[Mesh] OR child*[tiab] OR infant*[tiab] OR newborn*[tiab] OR neonate*[tiab] OR adolescent*[tiab] OR teen*[tiab] OR pediatric*[tiab] OR paediatric*[tiab]).

EMBASE: ('larynx edema'/exp OR 'endotracheal intubation'/exp OR (laryn* NEAR/3 (edema* OR oedema*)):ab,ti OR (stridor* AND (intubat* OR extubat* OR 'tracheal tube*')):ab,ti) AND ('glucocorticoid'/exp OR steroid*:ab,ti OR corticosteroid*:ab,ti OR glucocorticoid*:ab,ti OR prednisone:ab,ti OR prednisolone:ab,ti OR methylprednisolone:ab,ti OR dexamethasone:ab,ti OR cortisone:ab,ti OR hydrocortisone:ab,ti OR budesonide:ab,ti OR fluticasone:ab,ti OR ciclesonide:ab,ti OR triamcinolone:ab,ti OR beclomethasone:ab,ti OR flunisolide:ab,ti OR mometasone:ab,ti) AND (('juvenile'/exp OR 'pediatrics'/exp OR child*:ab,ti OR infant*:ab,ti OR newborn*:ab,ti OR neonate*:ab,ti OR adolescent*:ab,ti) AND teen*:ab,ti OR pediatric*:ab,ti OR paediatric*:ab,ti) AND [embase]/lim.

## Abbreviations

CI: Confidence interval
ICU: Intensive Care Unit
OR: Odds ratio
PRISMA: Preferred Reporting Items for Systematic Reviews and Meta-Analyses
RCT: Randomized controlled trial
RR: Risk ratio
