# Supplementary material for: Effectiveness of corticosteroids for post-extubation stridor and extubation failure in pediatric patients: a systematic review and meta-analysis
Source: Ann Intensive Care. 2020 Nov 18;10:155. doi: 10.1186/s13613-020-00773-6 (PMC7672172; doi:10.1186/s13613-020-00773-6)
Supplement: Supplementary file 1 — Additional file 1: Table S1. Summary of the characteristics of randomized controlled trials for assessment of post-extubation stridor and/or extubation failure. Table S2. Summary of the characteristics of observational studies for assessment of post-extubation stridor and/or extubation failure. Table S3. Quality assessment of observational studies. Table S4. Meta-regression analysis of post-extubation stridor and reintubation. [file 13613_2020_773_MOESM1_ESM.docx]

Table S1. Summary of the characteristics of randomized controlled trials for assessment of post-extubation stridor and/or extubation failure

| **Author** | **Year** | **Location** | **Multicenter** | **Type of ICU** | **Sample size (n)** | **Male (%)** | **Population** | **Mean age (month)** | **Corticosteroid regimen** | **Cumulative Equivalent dose of hydrocortisone (mg)** | **Observation period after extubation (h)** | **Mean time first dosing before extubation (h)** | |
| --- | --- | --- | --- | --- | --- | --- | --- | --- | --- | --- | --- | --- | --- |
| Ferrara et al[28] | 1989 | USA | Single | NICU | 60 | - | Infants | 0.22 | Dexamethasone: a single dose of 0.25mg/kg | 6.25 | 24 | | 0.5 |
| Tellez et al[32] | 1991 | USA | Two | PICU | 153 | - | <1, 1-5, and >5 yr | 30.0 | Dexamethasone: 0.5 mg/kg, every 6 hours for a total of six doses | 75 | 24 | | 9 |
| Courtney et al[29] | 1992 | USA | Single | - | 42 | 64.3 | Infants | - | Dexamethasone: 0.5 mg/kg, given in 3 doses every 8 hours | 37.5 | 24 | | 17 |
| Couser et al[27] | 1992 | USA | Single | - | 60 | - | Infants | 0.70 | Dexamethasone: 0.25mg/kg, 3 doses every 8 hours | 18.75 | 24 | | 4 |
| Anene et al[33] | 1996 | USA | Single | ICU | 66 | 56.1 | <5 yr | 3.5^a^ | Dexamethasone: 0.5 mg/kg, every 6 hour for six doses | 75 | 24 | | 9 |
| Harel et al[30] | 1997 | USA | Two | PICU | 23 | 52.2 | <10 yr | 34.6 | Dexamethasone: 0.5 mg/kg, given in 4 doses every 6 hours | 50 | - | | 6 |
| Doyle et al[26] | 2006 | Australia, New Zealand, Canada | ≥ 3 | - | 70 | 52.9 | Infants | 0.75^a^ | Dexamethasone: 0.15 mg/kg per day for 3 days, 0.10 mg/kg per day for 3 days, 0.05 mg/kg per day for 2 days, and 0.02 mg/kg per day for 2 days | 22.25 | 72 | | - |
| Cesar et al[31] | 2009 | Brazil | Single | PICU | 32 | - | <5 yr | - | Dexamethasone: 0.2mg/kg, every 6 h, during 24 hours of follow up | 20 | 24 | | 1 |
| Malhotra et al[11] | 2009 | India | Single | ICU | 60 | 63.3 | Children | 93.4 | Dexamethasone: 0.5mg/kg, 4 hours prior to planned extubation, at extubation, and 6 and 12 hours after extubation | 50 | 24 | | 4 |
| Drago et al[34] | 2015 | USA | Single | PICU | 35 | 51.4 | Aged 1 m to 18 yr | 71.8 | Methylprednisolone: loading dose of 2mg/kg and tapered over 14 days | 56.25 | - | | - |

Table S2. Summary of the characteristics of observational studies for assessment of post-extubation stridor and/or extubation failure

| **Author** | **Year** | **Location** | **Study design** | **Multicenter** | **Type of ICU** | **Sample size (n)** | **Male (%)** | **Population** | **Mean age (month)** | **Corticosteroids** | **Total number of events (stridor)** | **Estimated OR for stridor (95% CI)** | **Total Number of events (extubation failure)** | **Estimated OR for extubation failure (95% CI)** | **Adjusted covariate** | |
| --- | --- | --- | --- | --- | --- | --- | --- | --- | --- | --- | --- | --- | --- | --- | --- | --- |
| Lukkassen et al[35] | 2006 | Netherlands | Retrospective cohort study | Single | - | 60 | 40 | Aged 4 wk to 6 yr | 16.6 | Dexamethasone: 0.6-2 mg/kg/d, beginning at least 4 h prior to extubation and continued for 24 h | 9 | 0 (0 – 0.71) | 6 | 0 (0 – 1.29) | None |  |
| Saleem et al[36] | 2009 | Pakistan | Retrospective cohort study | Single | PICU | 51 | NA | Aged 4 wk to 5 yr | 18.1 | Dexamethasone: 0.5 mg/kg every 6 hours for 3 doses, beginning at 6-12 hours prior to extubation | 7 | 1.88 (0.27 – 21.75) | 4 | 0.68 (0.05 – 10.20) | Matched on age |  |
| Laham et al[37] | 2012 | USA | Prospective cohort study | Single | PICU | 319 | 61 | Aged 0 to 20 yr | 16 | Dexamethasone: 0.5 mg/kg every 6 hours | 69 | 1.01  (0.51-1.94) | 29 | 2.39 (0.99-5.62) | None |  |

Table S3. Quality assessment of observational studies

| **Study, Year** | Representativeness of the exposed cohort | Selection of the non-exposed cohort | Ascertainment of exposure | Demonstration that outcome of interest was not present at start of study | Comparability of cohorts on the basis of the design or analysis | Assessment of outcome | Was follow-up long enough for outcomes to occur? | Adequacy of follow-up of cohorts | Overall quality score |
| --- | --- | --- | --- | --- | --- | --- | --- | --- | --- |
| Lukkassen et al[35] | * | * | * | * |  | * |  | * | 6 |
| Saleem et al[36] | * | * | * | * |  | * |  | * | 6 |
| Laham et al[37] | * | * | * |  |  | * | * | * | 6 |

Table S4. Meta-regression analysis of post-extubation stridor and reintubation

|  | **Postextubation stridor** | **Reintubation** |
| --- | --- | --- |
| **Variable** | **Univariate regression coefficient (95% CI)^a^** | **Univariate regression coefficient (95% CI)^b^** |
| **Cumulative dose of corticosteroid equivalent to hydrocortisone** | -0.0092 (-0.037, 0.019) | 0.021 (-0.015, 0.057) |
| **Time from the first dosing to extubation** | -0.12 (-0.35, 0.11) | 0.0096 (-0.22, 0.24) |
| **Age^a^** | 0.086 (-0.17, 0.34) | 0.11 (-0.30, 0.52) |

^a^Median age and interquartile range were reported for Anene et al’[33]’s and Doyle et al[26]’s trials
