# Supplementary figures and images for: Effectiveness of corticosteroids for post-extubation stridor and extubation failure in pediatric patients: a systematic review and meta-analysis
Source: Ann Intensive Care. 2020 Nov 18;10:155. doi: 10.1186/s13613-020-00773-6 (PMC7672172; doi:10.1186/s13613-020-00773-6)

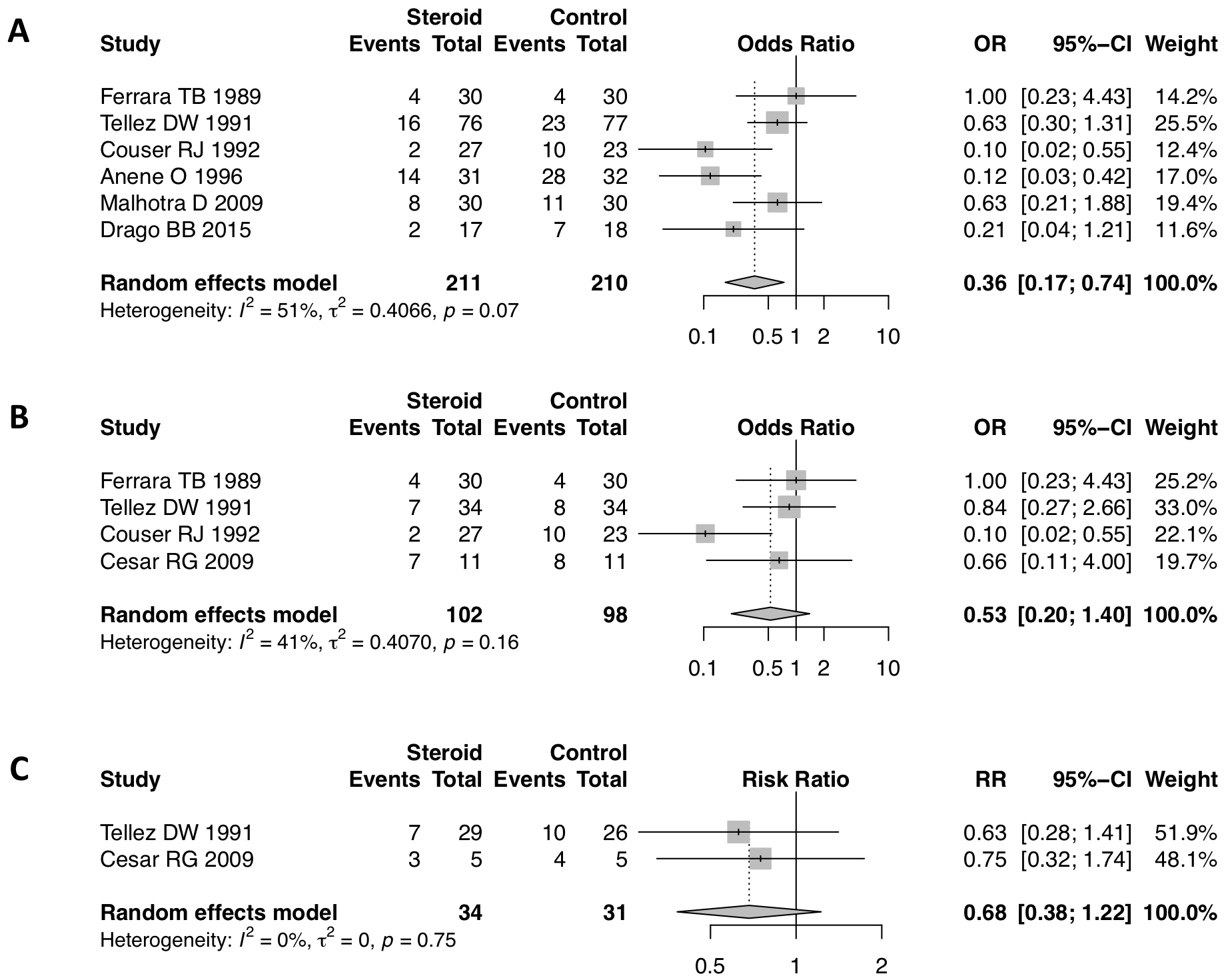

Supplement: Supplementary file 2 — Additional file 2: Figure S1. (A) Result of meta-analysis for effects of corticosteroids on incidence of stridor in randomized controlled trials. Results of meta-analysis for effects of corticosteroids on incidence of stridor/suspected upper airway obstruction among (B) infants and (C) pediatrics under five years old, not including infants in randomized controlled trials. An odds ratio (OR) less than 1 favors corticosteroid administration. [file 13613_2020_773_MOESM2_ESM.tiff]

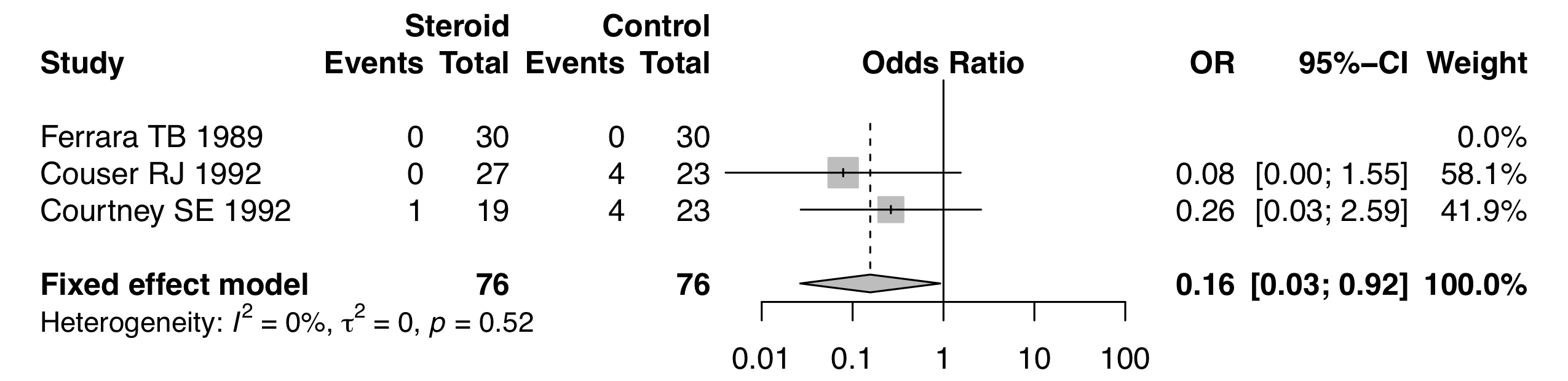

Supplement: Supplementary file 3 — Additional file 3: Figure S2. Results of meta-analysis for effects of corticosteroids on incidence of extubation failure among infants in randomized controlled trials. An odds ratio (OR) less than 1 favors corticosteroid administration. [file 13613_2020_773_MOESM3_ESM.tiff]
